# Insights into isoform-specific mineralocorticoid receptor action in the hippocampus

**DOI:** 10.1530/JOE-22-0293

**Published:** 2023-07-12

**Authors:** Carolina Gaudenzi, Karen R Mifsud, Johannes M H M Reul

**Affiliations:** Neuro-Epigenetics Research Group, Dorothy Hodgkin Building, University of Bristol, Bristol, United Kingdom

**Keywords:** mineralocorticoid receptor, glucocorticoid hormone, hippocampus, splice variants, isoforms, genomics, hypothalamic–pituitary–adrenal axis, stress

## Abstract

The mineralocorticoid receptor (MR) plays a critical role in the mammalian brain as a mediator of appropriate cellular and behavioural responses under both baseline and stressful conditions. In the hippocampus, the MR has been implicated in several processes, such as neuronal maintenance, adult neurogenesis, inhibitory control of the hypothalamic–pituitary–adrenal axis, and learning and memory. Because of its high affinity for endogenous glucocorticoid hormones, the MR has long been postulated to mediate tonic actions in the brain, but more recent data have expanded on this view, indicating that the MR elicits dynamic responses as well. The complexity of the diverse molecular, cellular, and physiological functions fulfilled by the human, rat and mouse MR could at least partially be explained by the existence of different isoforms of the receptor. The structural and functional characteristics of these isoforms, however, have remained largely unexplored. The present article will review the current knowledge concerning human, rat, and mouse MR isoforms and evaluate seminal studies concerning the roles of the brain MR, with the intent to shed light on the function of its specific isoforms.

## Introduction

The central actions of corticosterone and cortisol – the most abundant glucocorticoid hormones (GCs) in rodents and humans, respectively – are mediated by two intracellular steroid receptors: the glucocorticoid receptor (GR) and the mineralocorticoid receptor (MR) (Reul & de Kloet 1985, 1986, Reul *et al*. 1987). Both MRs and GRs are highly expressed in the hippocampus, a key limbic area of the brain which is critically involved in regulating adaptive responses to stressful challenges (Reul 2014). The hippocampus is also one of the few areas in the brain with an active neurogenic niche, generating newborn neurons across the lifespan (Kempermann 2002, Van Praag *et al*. 2002). Hippocampal MRs and GRs have been documented as participating in core functions of the hippocampus, but the exact mechanisms through which these receptors contribute to the functioning of this brain region remain elusive. Notably, GC levels in the brain are regulated by 11β-hydroxysteroid dehydrogenase enzymes (Holmes & Seckl 2006, Chapman *et al*. 2013), but this interesting subject is beyond the scope of this review paper.

MRs display an at least ten-fold higher affinity for cortisol and corticosterone than GRs and thus are highly occupied by hormones even at baseline hormone levels (Reul & de Kloet 1985, Arriza *et al*. 1987, Reul *et al*. 1987, Reul *et al*. 1990). In contrast, the GR has lower affinity for endogenous GCs, and, consequently, only the surges in hormone secretion that occur during the circadian peak or stressful situations lead to substantial receptor occupancy (Reul & de Kloet 1985, Reul *et al*. 1987, 1990). Therefore, it has long been postulated that the MR fulfils tonic functions that maintain brain homeostasis, whilst the GR is predominantly involved in phasic actions mediating stress- and circadian-related GC responses (Reul *et al*. 1987). More recently, however, this view has been adjusted. Using MR and GR chromatin immunoprecipitation (ChIP) on rat hippocampus chromatin, we found that, under baseline early morning (BLAM) conditions, MR binding to GC response elements (GREs) within the well-known GC-target genes *Fkbp5* (FK506 binding protein 5), *Per1* (Period 1) and *Sgk1* (Serum/GC-regulated kinase 1) was relatively low and increased significantly after an acute stressful challenge (Mifsud & Reul 2016). Thus, despite the high occupancy of MRs by hormone (Reul & de Kloet 1985) and the nuclear localisation of this receptor under BLAM conditions (Gesing *et al*. 2001), binding of the MR to GREs was muted. These observations clearly clashed with the concept of MR-mediated tonic effects on brain function. Recent ChIP-sequencing (ChIP-seq) analysis of MR interaction with the entire rat hippocampus genome led us to diversify our concepts on the functional role of the MR in this brain structure. We found that approximately 72% of MR peaks, corresponding to receptor-bound genomic regions, were responsive to acute stress conditions and circadian-induced changes, whereas 28% of peaks with significant MR binding did not respond to these physiological changes (Mifsud *et al*. 2021). The constant MR peaks may be functionally commensurate with tonically acting receptors. Responsive MR peaks, however, underwent a five- and seven-fold increase in MR binding following acute stress and at the peak of the circadian rhythm, respectively (Mifsud *et al*. 2021). These findings indicate that the MR, while certainly mediating tonic/constant actions of GCs, holds a substantial dynamic role as well.

Given such distinct differences (constitutive and/or dynamic) in the genomic binding profiles of the MR, c.f. the GR, this review seeks to provide an overview of the relevant literature to determine whether differential MR isoform expression could offer a plausible explanation for such diversity. The existence of different receptor isoforms that could mediate various functions has received relatively little consideration to date. Consequently, this review aims to provide a detailed overview of the characteristics of such isoforms in the human and rodent brain, specifically focusing on the hippocampus, to highlight how structural differences in the receptor molecule may contribute to the diversity of MR-dependent molecular, cellular, and behavioural processes. This review also discusses some seminal hippocampal MR studies where retrospective analysis has revealed that only some specific MR isoforms, rather than all receptor proteins, were targeted. Furthermore, this review summarises the progress that has been made regarding the molecular biology and functionality of the hippocampal MR, revealing its intriguing action and complexity, thereby lifting some but not all of its mysteriousness (Reul *et al*. 2000).

### The structural characteristics of the MR mRNA and protein

**Figure 1:**
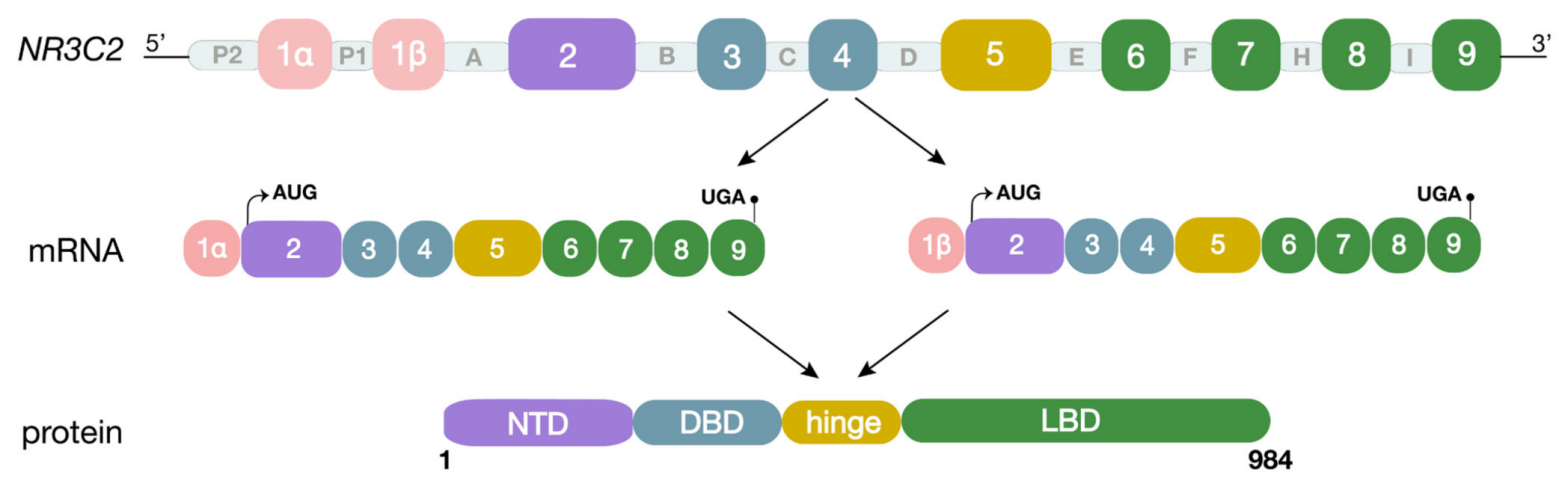
Schematic representation of the human *NR3C2* gene and its transcript and protein products. The human *NR3C2* gene comprises eight introns (A to I), as well as eight translated (2 to 9) and one untranslated exon. The latter includes two 5′-UTR, 1α and 1β, which are under the control of promoters P2 and P1, respectively. Alternative transcription of either 1α or 1β gives rise to two different mRNA species, α (left) or β (right). Exon 2 forms the NTD, exons 3 and 4 are translated into the DBD, exon 5 produces the hinge region, and exons 6 to 9 form the LBD. AUG/ATG, start codon; DBD, DNA-binding domain; LBD, ligand-binding domain; NTD, N-terminal domain; UGA, stop codon; UTR, untranslated region.

The MR is encoded by the *NR3C2* gene (Arriza *et al*. 1987) (Fig. 1). In humans, the first exon is variable (1α or 1β) and excluded from the translated sequence, which itself is the product of the remaining eight exons (Zennaro *et al*. 1995). These features also characterise the rat *Nr3c2* gene, which possesses three first-exon variants or 5′-untranslated regions (UTRs): 1α, 1β, and 1γ (Kwak *et al*. 1993). The exons located downstream of the UTRs encode for the different functional domains of the MR protein. Exon 2 gives rise to the N-terminal domain (NTD), which contains regions responsible for ligand-independent transactivation (Pascual-Le Tallec & Lombès 2005). Encoded by exons 3 and 4, the centrally located DNA-binding domain (DBD) (Zennaro *et al*. 2001) is able to recognise GREs located in promoter, intronic, or intergenic regions of downstream target genes (Pascual-Le Tallec & Lombès 2005). The last five exons form the ligand-binding domain (LBD) (Zennaro *et al*. 1995) which, in isoforms containing this region, is responsible for ligand-dependent transactivation (Zennaro *et al*. 2001). In the absence of ligand, an inactive conformation of the LBD is adopted through multiple contact sites with heat shock proteins (HSP90 and HSP70) (Binart *et al*. 1991, Bruner *et al*. 1997). Upon GC binding to the hydrophobic pocket within this domain, a nuclear localisation sequence (NLS) is exposed, which enables the MR to be translocated to the nucleus, thereby allowing the receptor to affect gene transcription (Lombès *et al*. 1994, Nishi *et al*. 2001).

### The structure and function of the MR splice variants and isoforms

The *NR3C2* gene transcript undergoes different splicing events, which result in a range of distinct transcriptionally and translationally derived MR products (Tables 1, 2, 3 and 4). The unique structural features that characterise each resulting variant have been hypothesised to lead to different transactivation abilities (Pascual-Le Tallec & Lombès 2005), which could modulate wildtype MR-mediated effects. Moreover, the MR protein isoforms could act independently of their wildtype counterpart in manners that have yet to be explored. Five major splice variants affecting the coding sequence and their associated protein isoforms have been identified in the human brain and are present in the Uniprot database. Analogous isoforms in both rats and mice are included for discussion (Table 4) (Bloem *et al*. 1995, Zhou *et al*. 2000, Zennaro *et al*. 2001, Guo *et al*. 2020). An additional splice variant (MRΔ6) has also been identified in mice (Lema *et al*. 2017) but is not yet listed on Uniprot. Several reports have described other MR receptor variants that derive from single nucleotide polymorphisms (SNPs), but these are outside the scope of this review and will not be included for discussion here.

### Canonical human, rat, and mouse MR

Alternative inclusion of exons 1α, 1β, or 1γ within the mature mRNA gives rise to the three respective MR splice variants α, β, and γ, with the latter one being exclusively found in rodents (Kwak *et al*. 1993), as exon 1γ is absent from the human *NR3C2* gene (Tables 1 and 2) (Zennaro *et al*. 1995). The location of the translation initiation site 2 base pairs downstream of the 5′ border of exon 2 leads 1α, 1β, and 1γ to remain untranslated, conferring the same open reading frame to all three splice variants. Therefore, translation of the α, β, and γ mRNA species generates the same canonical protein (MR isoform 1) in mice (mMR; UniProt ID: Q8VII8, 978 amino acids (aa), 106 kDa; A3KN90, 980 aa, 106 kDa), rats (rMR; UniProt ID: P22199-1, 981 aa, 106 kDa), and humans (hMR; UniProt ID: P08235-1, 984 aa, 107 kDa) and is conventionally considered to be the wildtype form of the MR protein in these species (Zennaro *et al*. 1995). For an overview of the characteristics of the MR splice variants and isoforms, as well as their respective database information, see Tables 1, 2, 3 and 4.

While the α, β, and γ splice variants have no effect on the final amino acid sequence, their 5′-UTR does seem to play a role in mRNA stability and, consequently, in the levels of MR translation (Zennaro *et al*. 1995). As a result, the differential expression of these three mRNA variants modulates overall MR concentration in a tissue-specific manner (Kwak *et al*. 1993, Zennaro *et al*. 1995, 1996, 1997, Vàzquez *et al*. 1998, Kang *et al*. 2009). In fact, Vàzquez and colleagues (1998) reported that the levels of α, β, and γ are regulated as a function of age in the rat hippocampus. Specifically, it was observed that expression of the α variant remains relatively constant in all hippocampal regions throughout development, and as such, the α variant is the most abundant mRNA species in the adult brain compared to the other two. On the other hand, the β and γ variants are expressed at higher levels than the α variant in early life, particularly in the CA2/3 and dentate gyrus (DG), respectively. After weaning from maternal care, the levels of the β variant decrease, while those of the γ variant remain higher even postweaning. While the exact significance of this differential expression pattern throughout the hippocampus is unclear, the authors hypothesised that the expression of these three mRNA species may be orchestrated by different promoter sequences that could determine translational efficiency or mRNA stability, thus ultimately controlling the levels of MR translation (Vàzquez *et al*. 1998). Considering that the correct timing and tissue specificity of gene expression are particularly fundamental for early development, Vázquez and colleagues surmised that the β and γ variants may be particularly important for early developmental processes. Specifically, because of its increased abundance during the first two weeks of life in the CA2 and CA3, the β variant could be associated with synaptogenesis and growth of commissural and associative terminal fields, which are processes that mostly occur during the first weeks of life in the CA2 and CA3. Similarly, the γ variant could be related to neurogenesis, as its expression is significantly higher in the developing DG during the first week of life, at which stage considerable levels of neurogenesis are observed (Vàzquez *et al*. 1998).

### The MRΔ5,6 splice variant: the MRΔ5,6 protein

Characterised by the deletion of exons 5 and 6, the *MR*Δ*5*,*6* splice variant displays a shift in the open reading frame that produces a premature stop codon located 107 base pairs downstream of the exon 4/intron D junction. The resulting protein, the hMRΔ5,6 isoform (UniProt ID: P08235-2, MR isoform 2), is shorter than the wildtype (706 aa, 75 kDa) as it lacks part of the hinge region as well as the entirety of the LBD. It gains a terminal 35-amino acid stretch that bears no homology with any other nuclear receptor sequence (Zennaro *et al*. 2001). The existence of this variant was ascertained in humans, where it was found to be highly expressed in the brain and the hepatic cell line SK Hep1, with substantial amounts present also in the lungs, kidney, heart, and lymphocytes. As this isoform was discovered in mouse kidneys as well (mMRΔ5,6, UniProt ID: D6RIL1, 698 aa, 74 kDa) (Zennaro *et al*. 2001, Lema *et al*. 2017), it is plausible that the MRΔ5,6 variant also exists in rats, but follow-up studies have yet to determine this (Tables 1, 2 and 3).

**Figure 2:**
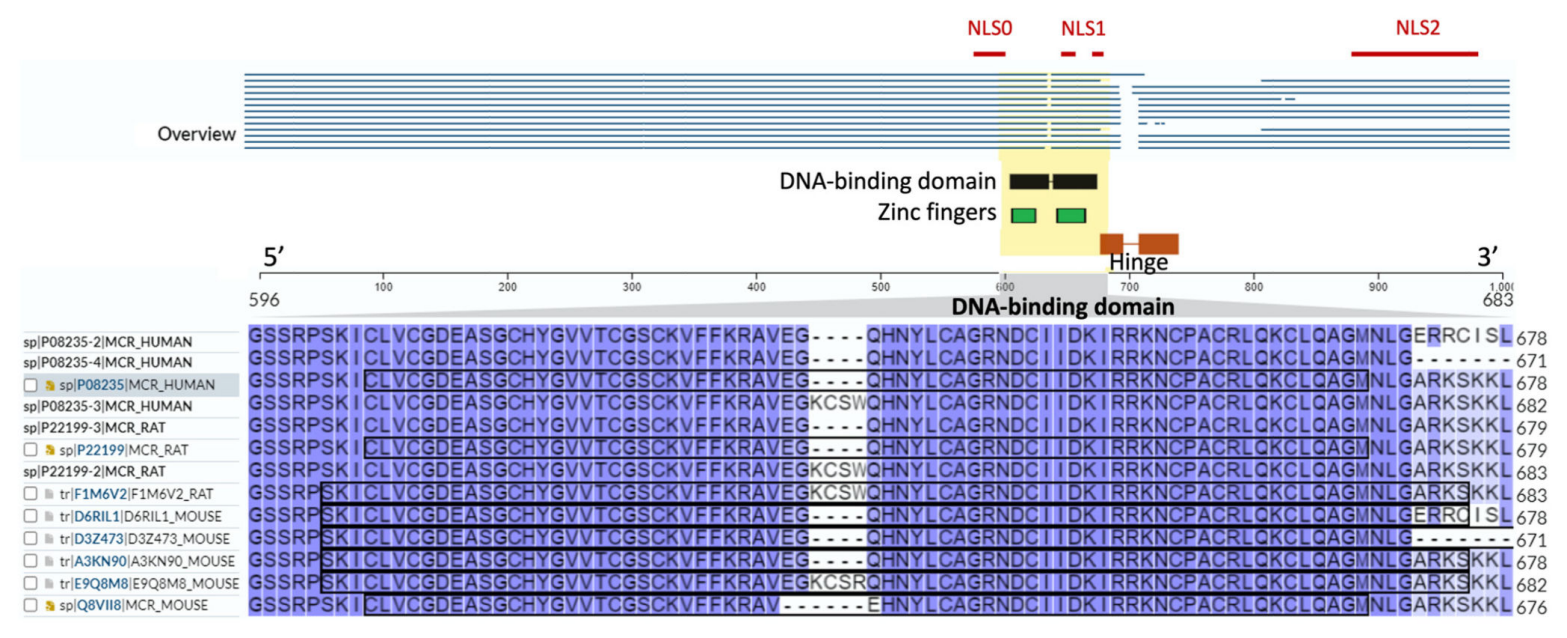
Sequence homology of the human, rat, and mouse MR (mineralocorticoid receptor; gene: *NR3C2*) protein sequences including the position of the NLS, DBD, zinc fingers, and hinge regions, and the 12 bp (4 amino acid (aa)) insert (The UniProt Consortium 2023). DBD, DNA-binding domain; NLS, nuclear localisation sequence.

The complete absence of the C-terminal region considerably affects the transcriptional activity of hMRΔ5,6, which, while retaining DNA-binding abilities almost identical to those of the wildtype protein, is unable to bind ligand, resulting in hormone-independent transcriptional properties (Zennaro *et al*. 2001). Interestingly, the presence of hMRΔ5,6 at an equal ratio to the wildtype hMR significantly enhanced the transactivational ability of the wildtype MR by up to 200% in rabbit RCSV3 cells, which are derived from kidney cortical collecting duct (Zennaro *et al*. 2001). The reason for this could be because, theoretically at least, hMRΔ5,6 retains the ability to form dimers, or potentially even multimers, with the ligand-activated wildtype hMR via the DBD (Liu *et al*. 1995, Rivers *et al*. 2019). As the hMRΔ5,6 lacks part of the NLS-1, located in the hinge region, and the entirety of the NLS-2, normally found in the LBD (Fig. 2), the potential of this isoform to dimerise or form multimers with wildtype hMR could thus facilitate its nuclear localisation. This ‘hetero’-dimerisation could enhance the transcriptional ability of the wildtype MR by increasing the number of receptors interacting with the DNA. In contrast, the presence of this isoform did not appear to affect the transcriptional activity of the wildtype MR in murine renal cells (Lema *et al*. 2017). More studies are therefore required to determine if such differences are cell- or species-specific, or a factor of circumstance (Tables 1, 2, 3 and 4).

### The MRIn12 splice variant: the MR+4 protein

Studies by Bloem and colleagues (Bloem *et al*. 1995) in both humans and rats have revealed the existence of a splice variant, *MRIn12*, that contains a 12-nucleotide insertion within exon 3, between codons for amino acids 634 and 635. Such in-frame insertion, deriving from the use of an alternative splice site between exon 3 and intron C, generates a protein, human MR+4 (hMR+4; UniProt ID: P08235-3, 988 aa, 107 kDa, MR isoform 3) and rat MR+4 (rMR+4; UniProt ID: P22199-2, 985 aa, 107 kDa, MR isoform 2), with four additional amino acids between the two zinc fingers of the DBD region (Bloem *et al*. 1995) (Fig. 2). According to database information, this isoform is also present in mice (mMR+4; UniProt ID: E9Q8M8, 984 aa, 107 kDa), but studies aimed at detecting the *MRIn12* mRNA splice variant in mice were unsuccessful (Rivers *et al*. 2009).

The insertion that characterises the MRIn12 transcript was hypothesised to distort the orientation of the second zinc finger, possibly causing a reduction in DNA binding affinity and/or dimerisation (Bloem *et al*. 1995), but to our knowledge, no further studies have tested this idea *in vivo*. This explanation seems plausible, however, considering that the DBD-located human G633R missense mutation of the *NR3C2* gene results in diminished maximal transactivation of a luciferase reporter gene under the control of two consensus GREs upstream of a TATA box. This mutation is located at the point of the MRIn12 insertion. Even though its DNA binding affinity is not affected, the R633 mutant receptor displayed an altered *in vitro* dissociation kinetics from a consensus GRE as, when incubated with a competitor (i.e. unlabelled oligonucleotides), it remained bound to GRE oligonucleotides for longer than the wildtype hMR. In addition, the mutation appears to affect the subcellular localisation of the R633 mutant protein as demonstrated by the fact that, even in the absence of hormone, the enhanced green fluorescence protein-linked mutant was almost entirely found in the nucleus or both in the nucleus and cytoplasm of transiently transfected RCSV3 cells, and the addition of aldosterone resulted in a faster translocation to the nucleus compared to that of the wildtype MR (Sartorato *et al*. 2004) (Tables 1, 2, 3 and 4). This distortion theory was challenged by Wickert *et al*. 2008 who used protein modelling to predict the consequence of the insertion and found that it led to ‘a hypothetical structure which is comparable to the MRwt protein’, concluding that the MR+4 is not likely to be ‘restricted in function’ (Wickert *et al*. 2008).

Following the discovery of *hMRIn12* mRNA in human white blood cells, where it is found at a higher abundance than the wildtype (Bloem *et al*. 1995), *MRIn12* mRNA has also been shown to be expressed in the human brain (Wickert *et al*. 1998). In a follow-up study, Wickert and colleagues found increased expression of *hMRIn12* mRNA in temporal lobe tissue samples obtained from patients with epilepsy compared with control brain tissues samples removed to facilitate tumour access (frontal/parietal/temporal lobe) but showing no tumour invasion (as assessed through neuropathological examination). This could implicate this variant in the pathology of epilepsy although future studies should take care to rule out confounding factors potentially influencing splice variant ratio such as brain region, effects of medication, etc (Wickert *et al*. 2008).

The *rMRIn12* mRNA was identified in the brain, heart, kidney, and liver. In rats, it was found to be less abundant than the wildtype MR in all tissues, with the exception of the liver, where it was expressed at comparable levels to the wildtype (Bloem *et al*. 1995, Rivers *et al*. 2009). Interestingly, overexpression of cytotoxic granule-associated RNA-binding proteins TIA1 and TIAL1, proteins known to act as splicing regulators, increased the expression of the *rMRIn12* mRNA variant in COS-1 cell expressing rat MR minigenes, whilst small interfering RNA-mediated knockdown of TIA1/TIAL1 abolished *rMRIn12* mRNA expression completely, suggesting a role for these proteins in the mechanism of alternative MR splicing (Rivers *et al*. 2009).

### The MRΔ5 splice variant: the MRΔ5 protein

Initially cloned by Arriza and colleagues, who reported an MR mRNA lacking nucleotides 2237 to 2587 (Arriza *et al*. 1987), the *hMR*Δ*5* splice variant was later confirmed in experiments that demonstrated the identity of the deleted fragment as exon 5 (Zennaro *et al*. 2001). The resultant receptor protein, in humans MRΔ5 (hMRΔ5; UniProt ID: P08235-4, 867 aa, 94 kDa, MR isoform 4), lacks amino acids 672–788, which corresponds to the whole hinge region, co-activator binding region, and the N-terminal part of the LBD (Zennaro *et al*. 2001). The *hMR*Δ*5* splice variant was found in human lungs, kidneys, heart, and hippocampus, but it was absent in hepatic cell lines. An analogous isoform has been reported for rats (rMRΔ5; UniProt ID: A0A8I6G6P1; 868 aa, 94 kDa) (Arriza *et al*. 1987) and mice (mMRΔ5; Uniprot ID: D3Z473, 867 aa, 94 kDa), although little experimental data for this isoform in rodents exist (Tables 1, 2, 3 and 4).

The in-frame deletion that characterises the *MR*Δ*5* transcript has been hypothesised to alter the three-dimensional structure of the resulting protein product, which, similar to hMRΔ5,6, exhibits diminished ligand-binding activity (Guo *et al*. 2020). Conversely, unlike the dominant positive effect exerted by hMRΔ5,6, hMRΔ5 seems to mediate negative actions on wildtype MR functioning. In fact, in placental and umbilical artery tissues of patients with preeclampsia, in which *MR*Δ*5* mRNA was found to be overexpressed, the typical MR downstream genes *FKBP5* and epidermal growth factor receptor, were downregulated (Guo *et al*. 2020). Impaired ligand binding or nuclear translocation abilities due to the missing hinge region were hypothesised to be plausible explanation for the negative outcomes mediated by this isoform (Guo *et al*. 2020).

### The MRdel10 splice variant: the del10MR protein

The *MRdel10* splice variant was identified in both humans and rats by Zhou and colleagues, who reported an MR mRNA species lacking ten base pairs in the C-terminal LBD region. This deletion, comprising nucleotides 2347 to 2356, generates a frameshift that results in a premature stop codon (Zhou *et al*. 2000). The derived rat del10MR isoform (rdel10MR; UniProt ID: P22199-3, 807 aa, 86 kDa, rat isoform 3) has most of the LBD missing, but retains an important co-activator binding region (779–782 aa) before diverging from the wildtype receptor at amino acid 783, where a unique 24-residue stretch concludes the protein product (Zhou *et al*. 2000). The expression of MRdel10 mRNA was detected in the rat hippocampus, hypothalamus, and all the peripheral tissues where the wildtype rMR is expressed. Specifically, the transcript abundance of the deletion variant was estimated to be 6% and 4% of the wildtype MR in the rat kidney and hippocampus, respectively. This isoform was also detected in human kidneys (Zhou *et al*. 2000), but sequence details were not made available. Considering that the human isoform is not currently listed on Uniprot, specific interspecies comparison of isoform structure is not possible.

### Differential splicing mechanisms

Whilst not much is known regarding the specific molecular pathways leading to the generation of most of these MR isoforms, there is evidence that an RNA-binding protein by the name of human antigen R (HuR) is involved in the splicing events that result in the expression of the *mMRΔ6* mRNA in renal cells (Lema *et al*. 2017). HuR is expressed throughout the body and is a member of a family of RNA-binding proteins that includes the neuron-specific proteins HuB, HuC, and HuD, which have important roles in neuronal differentiation and neurogenesis (Wakamatsu & Weston 1997, Hilgers 2022). Hu proteins can mediate splicing events (both positive and negative) through spliceosome modulation and/or chromatin modification. The function of Hu proteins during splicing events is location- and gene-specific. In some cases, binding of Hu to AU-rich target elements can directly affect the interaction between the spliceosome and splice sites, resulting in either exon skipping or exon inclusion, such as in the cases of neurofibromatosis type 1 (Nf1) and Human Antigen D (HuD), respectively (Zhu *et al*. 2008, Wang *et al*. 2010). Hu binding is also capable of locally inhibiting the action of histone deacetylase 2, leading to hyperacetylation of local chromatin and increased transcriptional elongation rates, which in turn can promote exon skipping (Zhou *et al*. 2011). Clearly, elucidation of the molecular mechanisms underlying differential splicing of MR RNA requires further investigation.

### Functional studies on the brain MR

Early work to determine the neuroanatomical localisation of the MR through cytosol radioligand binding assays (Reul & de Kloet 1985) or *in vitro* autoradiography (Reul & de Kloet 1986) relied heavily on the presence of the LBD within the receptor molecule. It is important, therefore, to note that these early studies would have failed to detect the truncated MR isoforms lacking this region (hMRΔ5,6; hMRΔ5; hMRΔ6; del10MR). Likewise, the advent in the late 1980s/early 1990s of commercially available antibodies targeting specific regions of the MR protein, such as the popular H10E4C9F anti-idiotypic antibody, or polyclonal antibodies directed against the NTD (N17 sc6860, H-300 sc11412; sc: Santa Cruz) or, later, against the C-terminal domain (CTD) (C-14 sc6861), predated the discovery of some of the MR isoforms mentioned earlier. Therefore, what was, at the time, considered as a comprehensive documentation of general MR actions may in fact be limited to the function of the specific isoforms targeted by each antibody. In this section, we review seminal papers documenting the critical role of the MR in neuronal development and responses to stress, highlighting instances where isoform specificity may explain some of the diverse nature of MR actions in the brain.

### MR-mediated effects in the hippocampus

The MR is highly enriched in the hippocampus, specifically in the DG and CA1–3 regions. Early studies to determine the role of GC action in the rat hippocampus employed adrenalectomy (ADX) to remove endogenous GCs. Within days, ADX resulted in significant neuronal cell death, which was exclusively observed in the granular cell layer of the DG regardless of age, sex, and rat strain (Sloviter *et al*. 1989, 1993). Interestingly, this neuronal cell death was only noticed in those cases where clinical signs of adrenal insufficiency (poor weight gain, low sodium, and high potassium in plasma), which confirmed the ‘completeness’ of ADX, were present. Either ‘incomplete’ ADX or GC hormone originating from other sources (e.g. ectopic adrenal cells) did not lead to hippocampal damage in some ‘ADX’ animals (Sloviter *et al*. 1989). This was an interesting finding, as it implied that even small amounts (~15 ng/mL, McNeill *et al*. 1991) of circulating GCs were capable of protecting against neuronal cell death in the DG. Given that the MR had already been shown to exhibit a very high binding affinity for such low levels of corticosterone cf. the GR (Reul & de Kloet 1985), it stood to reason that corticosterone was likely to be the corticosteroid hormone acting via this receptor in the DG to exert this protective effect. Follow-up studies using specific MR agonists and antagonists further implicated the MR as playing a role in mediating this protective function, which is dependent on a functional LBD (Almeida *et al*. 2000, Macleod *et al*. 2003, Crochemore *et al*. 2005).

A similar widespread cell loss occurs in the neonatal hippocampus of rodents during the postnatal period, when circulating corticosterone levels fall to barely detectable levels (10–30 ng/mL, 6–48 h after birth approximately until the post-natal day (PDN) 15) (Meaney *et al*. 1985). Interestingly, this is also a period of enhanced postnatal neurogenesis, resulting in the structural formation of the DG (Altman & Bayer 1990). Manipulation of corticosterone availability during this period reveals differential effects on neuronal proliferation and apoptosis during DG development (Gould *et al*. 1991). Longer MR isoforms with an intact LBD are expressed in the rat hippocampus during this early postnatal period (Van Eekelen *et al*. 1991), with protein levels starting out low/undetectable at PND0 but increasing rapidly to adult levels by PND8 (Rosenfeld *et al*. 1993). However, no morphological differences were observed in the developing hippocampus/DG structures at PND8 between MR gene-deleted (MR^−/−^) and wildtype mice (Gass *et al*. 2000). It remains unclear, therefore, if the MR is mediating these corticosterone-induced effects on neurogenesis/apoptosis during early DG development.

The DG is one of the few places within the brain that continues to produce new neurons into adulthood. This so-called ‘adult’ neurogenesis is distinct from the process of embryonic/postnatal neurogenesis discussed earlier. Adult neurogenesis is a complex process comprising several stages that occur over 6–8 weeks in rats, and as such, it is highly susceptible to regulation by external factors (Kempermann *et al*. 2004). In adult MR^−/−^ mice, the density of granule cells was overall diminished, and the number of Mib1-positive cells, which is a marker of proliferation, was decreased by 65% compared to wildtype littermates, implicating the MR in ongoing adult-induced neurogenesis (Gass *et al*. 2000). Activation of the MR by aldosterone administration to adult ADX rats for 1 month increased the number of seven-day-old neurons compared to ADX + saline and sham-ADX animals. In addition, approximately half of the neurons also expressed the neuronal marker Tuj1 indicating neurogenic differentiation (Fischer *et al*. 2002). In contrast, Wong and Herbert showed that corticosterone replacement prevented the ADX-induced increase in the number of 4-day-old DG neurons (representative of the activation/proliferative stage), which was partially reversed by administration of the MR antagonist spironolactone (Wong & Herbert 2005). Similarly, Montaron and colleagues showed increased proliferation of 3-day old DG neurons in 5-day ADX rats, an effect that was prevented by aldosterone administration. Interestingly, the levels of the polysialylated isoform of the neural cell adhesion molecule (PSA-NCAM, a neuronal differentiation marker) did not change following aldosterone administration (Montaron *et al*. 2003). The apparent differential effects of MR agonists and antagonists on neuronal development in the abovementioned studies may be explained by the following reasons. First, neurogenesis comprises several stages, including proliferation, neuronal differentiation, migration, maturation, and survival, which, in rats, occur over a 6- to 8-week period. These studies analysed different stages of neurogenesis (differentiation vs proliferation), implying that experimental timing should be considered when interpreting results. Furthermore, there were also key experimental differences in the studies regarding ADX delay time, neuronal labelling protocols, and rat strain used. Nevertheless, it is obvious that manipulations of MR activity have clear effects on various aspects of neurogenesis. Specifically, such effects appear to be dependent on the LBD, suggesting that ligand-dependent MR isoforms are primarily involved in these neurogenic functions. This assumption is further indirectly supported by the observation that, when the truncated *hMR*Δ*5* mRNA is overexpressed in human umbilical vein endothelial cells, proliferation and migration, both essential components of neurogenesis, are prevented (Guo *et al*. 2020).

Whilst the MR has long been implicated as an important regulator of neurogenesis, as evidenced by the abovementioned studies and several others that, due to space limitations, have not been mentioned here, the exact molecular mechanisms through which the MR achieves such effects remain unclear. Recently, we performed ChIP in combination with next-generation sequencing (NGS) on rat hippocampus chromatin samples using an MR antibody targeting the NTD (sc-11412 Santa Cruz), a region present in all rat MR isoforms (Mifsud *et al*. 2021). A subset of genes (~30%) showed constant MR binding under all physiological conditions studied, that is, genomic MR binding did not change after acute stress or circadian variation. Furthermore, gene ontology and ingenuity pathway analysis (IPA) uncovered a significant association between the MR and ciliary genes encoding proteins involved in cilia structure and function. Further bioinformatic analysis revealed that a high proportion of ciliary genes bound the MR constitutively. Primary cilia are small hairlike protrusions on cells that have critical roles in neuronal stem cell proliferation and maintenance (Breunig *et al*. 2008, Amador-Arjona *et al*. 2011). Due to the discontinuation of the Santa Cruz MR antibody used for the MR ChIP-seq in the Mifsud *et al*. (2021) study, alternative antibodies were used in subsequent analyses. Interestingly, constitutive binding of the MR to cilia-related genes was only found to be significantly enriched when using an MR antibody directed against the NTD (ab97834, Abcam), but not with a C-terminal directed MR antibody (ab64457, Abcam), potentially implicating ligand-independent MR isoforms in constitutive binding to the DNA (Mifsud *et al*. 2021). Additional work is ongoing to determine if this hypothesis holds any functional relevance.

IPA also revealed a significant association between MR and neuronal differentiation. This finding is relevant when considering that the presence of the sex steroid progesterone in the culture medium has been empirically determined to be a critical requirement for the differentiation of stem cells (Bottenstein & Sato 1979). Because progesterone can act as an MR agonist under certain conditions, we sought to verify the requirements for steroid receptor activation during the differentiation process. Using human fetal neuronal progenitor cells (hfNPCs), we discovered that principal MR agonists, namely corticosterone and deoxycorticosterone, were both capable of inducing differentiation of hfNPC in the absence of progesterone (Mifsud *et al*. 2021), thus supporting our IPA data linking MR activity with neuronal differentiation. Furthermore, we showed that expression of the MR (using ab64457, which targets the CTD) was only observed in differentiated neurons, but not in proliferating cells prior to differentiation. Furthermore, the lack of any MR agonist in the culture media completely abrogated neuronal differentiation. Finally, blocking the MR with spironolactone significantly reduced the nuclear localisation of the MR, diminished cilia formation, and prevented the differentiation of hfNPCs (Mifsud *et al*. 2021). Whilst further studies are required to unravel the relationship between the specific MR isoforms, cilia development, and neurogenesis, these findings indicate that steroid-inducible MR isoforms are required for successful neuronal differentiation (Mifsud *et al*. 2021).

### MR action and function associated with circadian variation and stress

#### Genomic actions

Historically, the genomic action of the MR has been associated with the maintenance of tonic responses to GCs, whilst the GR was thought to mediate the genomic effects of elevated GC levels (Reul & de Kloet 1985). We first provided evidence that binding of the MR, as well as that of the GR, was increased by stress and the circadian rise in GCs at certain well-known GC-dependent genes (*Fkbp5, Per1, Sgk1*) within the rat hippocampus (Mifsud & Reul 2016, 2018). This was supported by an NGS study on the hippocampus of ADX rats, which showed that corticosterone administration increased the binding of the MR to a significant number of GC-dependent genes (van Weert *et al*. 2017). Furthermore, we showed that ADX indeed completely prevented hippocampal MR binding to *Fkbp5, Per1, Sgk1*, and *Klf* (Krüppel-like factor) genes in baseline and acutely stressed rats (Kennedy *et al*. 2020, 2023). We also provided strong evidence supporting a role for the formation of MR:GR heterodimers in the transcriptional activation of GC-dependent genes (Mifsud & Reul 2016, 2018). These studies, together with the emergence of NGS data (Mifsud *et al*. 2021), have shown that the MR, even though already highly occupied by ligand even at baseline levels of GC hormone, plays a very active role in mediating the genomic effects of activational physiological states like stress and circadian peak conditions. Indeed, such physiological states significantly increase the binding of the MR at over 70% of MR-bound genes (Mifsud *et al*. 2021). MR ChIP was performed in both NGS studies (van Weert *et al*. 2017, Mifsud *et al*. 2021) using the now discontinued Santa Cruz sc11412 N-terminus-directed MR antibody, which targets all three rat isoforms. Different MR isoforms, however, could give rise to differential three-dimensional structures, possibly affecting antibody affinity. Key findings have since been validated using a number of MR antibodies targeting both the N- (ab97823, Abcam; 21854-1-AP, SAB2104689, Sigma) and C-terminal (ab64457, Abcam) domains. Interestingly, whilst the majority of stress- and circadian-induced MR binding was detected in genes that also bound the GR, there was a subset of genes that bound the MR in the absence of significant GR binding (Mifsud *et al*. 2021). Further work by Mifsud and colleagues has identified the Regulatory Factor X transcription factors as potentially co-localising with constitutive MR binding at many loci within the DNA in the absence of a GRE (Mifsud *et al*. 2021). More research is required to determine whether different MR isoforms are playing a role in these distinct genomic actions under various physiological conditions. In addition, further studies should explore which MR isoforms interact with the genome as a homodimer, heterodimer with GR, or in conjunction with alternate transcription factors.

#### Non-genomic actions

Repeated observations of the speed with which some MR-dependent behavioural adaptations ensue have indicated that genomic actions are not the only signalling cascades initiated by the MR. Therefore, fast-acting non-genomic effects of rising GCs had already been hypothesised when Karst and colleagues described these effects in the rat hippocampus, where bovine serum albumin-conjugated corticosterone, unable to cross the membrane, induced a 60% increase in miniature excitatory post-synaptic current (mEPSC) frequency. This indicated that a presynaptic membrane-localised MR may facilitate the probability of glutamate release, but only in the presence of high levels of circulating corticosterone (Karst *et al*. 2005). The dependence of these effects on corticosterone would suggest one of the ligand-binding isoforms of the MR to be involved in these non-genomic processes. Recent work by Karst and colleagues, however, failed to identify membrane-localised MRs in dendritic, post-synaptic, and pre-synaptic regions of primary rat hippocampal neurons. Interestingly, three different NTD-directed MR antibodies were employed (Karst *et al*. 2022), indicating that none of the MR isoforms that have so far been identified are responsible for the observed increase in mEPSC frequency. However, at this stage, technical issues in the detection of the membrane MR cannot be excluded.

### The role of the MR in HPA axis regulation and clinical significance

The hippocampal integrity and plasticity maintained by the MR are particularly important given the suppressive influence of the hippocampus on the activity of the hypothalamic–pituitary–adrenal (HPA) axis, and thus, on the speed, magnitude, and termination of the stress response (Joëls *et al*. 2008). MR-mediated excitatory outputs from the hippocampus impinge on GABAergic (inter)neurons in the bed nucleus of the stria terminalis and surrounding the hypothalamic paraventricular nucleus, thus modulating the activity of the parvocellular neurons involved in the synthesis of corticotropin-releasing hormone (Herman *et al*. 1989, 1992, 1995). Consequently, blocking of the MR with the MR antagonists RU28318 or spironolactone diminishes such inhibitory tone, thereby allowing for augmented HPA axis activation (Ratka *et al*. 1989, Gesing *et al*. 2001, Mifsud *et al*. 2021). Given the dependency of these results on antagonist binding, it is plausible that the MR-mediated negative feedback on the HPA axis is largely due to the ligand-binding isoforms of the receptor.

This role of the MR in preventing HPA axis hyperactivity by mediating tonic inhibitory mechanisms is clinically important when considering that a significant portion of depressed patients shows increased cortisol levels (Gold *et al*. 1980, Heuser *et al*. 1994, Høifødt *et al*. 2019) due to impaired inhibitory control of the HPA axis (Holsboer 2000, Pariante & Lightman 2008). The involvement of the MR in the pathophysiology of depression is suggested by the fact that MR mRNA expression in the hippocampus of patients suffering from major depressive disorder is lower than that observed in healthy subjects (Klok *et al*. 2011*a*). Furthermore, antidepressant treatment not only enhances the expression and consequent binding capacity of the MR in the rat hippocampus (Brady *et al*. 1991, Seckl & Fink 1992, Reul *et al*. 1993, 1994, Yau *et al*. 1995) but also causes a dampening of HPA axis activity (Reul *et al*. 1993, 1994). In patients suffering from depression who were treated with escitalopram, concomitant 3-week administration of the MR agonist fludrocortisone led to a faster response to the antidepressant treatment compared to patients who received the MR antagonist spironolactone or placebo (Otte *et al*. 2010). Taken together, these findings suggest that a functioning MR is required for the clinical improvement of depression, possibly by restoring inhibitory control of the HPA axis.

Concerning the specific MR isoforms that could be involved in this process, in both their studies, Reul and colleagues employed methods that detected the longer, ligand-binding isoforms of the MR (rMR, rMR+4) (Reul *et al*. 1993, 1994). This, in addition to the positive modulatory effects of agonist binding on clinical depression, implicates the LBD of long MR isoforms for the functional attenuation of the HPA axis mediated by antidepressants.

Stress, which has been repeatedly implicated in the development of mental disorders like major depression, results in an inhibition of adult neurogenesis in the hippocampal DG. This and other observations, such as that antidepressant treatments positively influence adult DG neurogenesis, have led to the hypothesis that this cellular process is involved in the development and treatment of depression (Santarelli *et al*. 2003, Henn & Vollmayr 2004, Holick *et al*. 2008, Schoenfeld & Cameron 2015). Considering the positive effects of the MR on multiple stages in the neurogenesis process, this receptor could play a dual role in the pathophysiology of depression not only through its suppressive effects on the HPA axis but also through its influence on neurogenesis. In this respect, it is of interest to mention that hippocampal MR has been shown to be very sensitive to long-term antidepressant drug treatment (Reul *et al*. 1993, 1994). As previously mentioned, its longer, ligand-binding isoforms could be responsible for these effects, even though it should not be excluded that the non-ligand-binding protein variants may participate in these processes in some yet undiscovered manner.

### Behavioural adaptations and learning

Downregulating and upregulating manipulations of MR expression in rodent models have shown that the performance of the MR at the cellular level is often associated with specific behavioural adaptations (Berger *et al*. 2006, Ferguson & Sapolsky 2007, Lai *et al*. 2007, Brinks *et al*. 2009). Similarly, studies on SNPs of the *NR3C2* gene have highlighted that the molecular actions of the MR influence human behaviour as well (De Rijk *et al*. 2006, Bogdan *et al*. 2010, Klok *et al*. 2011*b*, Van Leeuwen *et al*. 2011). This point is further supported by the implication of the MR in the process of strategy switching under stressful circumstances. In these instances, a dorsal striatal-mediated learning system, based on habits, becomes increasingly adaptive because, although more rigid, it is simpler and more rapid than the standard hippocampal-based spatial learning method, which allows for more complex cognition but is concomitantly demanding and slower (Schwabe *et al*. 2007). Therefore, when stress persists, the selection of a faster and less costly strategy has been reported to be generally more favourable for successful coping, as this is able to preserve optimal memory performance (Schwabe *et al*. 2013). The MR is thought to be a mediator in this process, as demonstrated by the fact that naïve mice pre-treated with spironolactone maintained a hippocampal-based approach that resulted in impaired learning and, consequently, suboptimal performance in learning tasks when compared to animals that employed a striatal method (Schwabe *et al*. 2010). These findings have been replicated in humans, where pharmacological blockade of the MR through spironolactone prevented strategy shifting (Schwabe *et al*. 2013). The dependency of these results on receptor blockade via an antagonist could once again be reflective of the involvement of the longer, ligand-dependent receptor isoforms in the process of strategy shifting. However, the different MR isoforms have not been directly investigated in relation to their potential roles in rodent and human behaviour. Moreover, the molecular/genomic basis of these MR-mediated behavioural effects is currently unknown.

## Summary

This review highlights that, at present, our knowledge concerning the involvement of specific isoforms in MR-mediated actions is still limited. In addition, the available literature on the topic is biased towards investigating the function of MR isoforms with intact LBDs. The absence of physiological effects after the administration of MR agonists or antagonists is often interpreted as exonerating the MR from the process being investigated when, in fact, this could implicate the truncated MR isoform. More focused methods, together with the development of antibodies directed at the unique regions of the different isoforms, are required. An increased understanding of the potentially different functions of the MR isoforms could have profound implications for our knowledge of critical processes, such as neurogenesis, neuroplasticity, and physiological and behavioural stress responses, in which the MR is thought to be involved. Furthermore, novel functions of the MR might also be uncovered.

## Figures and Tables

**Table 1:** Summary of the characteristics of the human mineralocorticoid receptor (hMR) mRNA splice variants.

Transcript nomenclature		Characteristics		Tissue expression		Abundance		Literature
α, β		Wildtype transcript		Expressed in aldosterone target tissues and the hippocampus		The wildtype mRNA species comprise >90% of total MR mRNA in brain tissues		Zennaro *et al.* (1997), Wickert *et al.* (2008)
hMRΔ5,6		Deletion of exons 5 and 6; lacks base pairs 2237–2732		Highly expressed in the hippocampus and in hepatic cell lines, lower levels in the colon		The hMRΔ5,6 to wildtype MR ratio is five-fold higher in the kidney than in the heart, with intermediary levels in the brain and liver		Zennaro *et al.* (2001)
hMRIn12		12-base pair insertion in the DBD		Existence confirmed in white blood cells		The insertion variant shows a slightly higher abundance compared to the wildtype MR in white blood cells		Bloem *et al.* (1995)
hMRΔ5		Deletion of exon 5; lacks base pairs 2237–2587		Expressed in hippocampus, heart, lungs, kidneys, and all other epithelial tissues except for hepatic cell lines		Not studied		Arriza *et al.* (1987), Zennaro *et al.* (2001), Guo *et al.* (2020)
hMRdel10		10-base pair deletion of base pairs 2347–2356		Not studied		Not studied		Zhou *et al.* (2000)

**Table 2:** Summary of the characteristics of the rat mineralocorticoid receptor (rMR) mRNA splice variants.

Transcript nomenclature		Characteristics		Tissue expression		Abundance		Literature
α		Wildtype protein		Constant after birth and predominant in adulthood		30% of total MR mRNA		Vàzquez *et al.* (1998), Kwak *et al.* (1993)
β		Wildtype protein		Highest during early life in the CA2/3; levels of expression decline afterwards		<30% of total MR mRNA		Vàzquez *et al.* (1998), Kwak *et al.* (1993)
γ		Wildtype protein		Highest during early life in the dentate gyrus; levels of expression decline afterwards		~3% of total MR mRNA		Vàzquez *et al.* (1998), Kwak *et al.* (1993)
rMRIn12		12-base pair insertion in the DNA-binding domain		Expressed in the liver, brain, heart, kidneys, and adrenal gland		Less abundant than the wildtype in all tissues except for the liver		Bloem *et al.* (1995)
rMRdel10		10-base pair deletion of base pairs 2347–2356		Present in all tissues in which the wildtype is present (kidney, aorta, colon, heart, hippocampus, hypothalamus)		rMRdel10 is 6% of the wildtype MR in the kidney and 4% in the hippocampus		Zhou *et al.* (2000)

**Table 3:** Summary of the characteristics of the mineralocorticoid receptor (MR) protein isoforms.

		Length										
Isoform		Human		Rat		Mouse		Characteristics		Transcriptional activity		Literature
Wildtype		984 (107 kDa)		981 (106 kDa)		980 (106.6 kDa; A3KN90); 978 (106.4 kDa; Q8VII8)^b^		Wildtype protein		Enhances or decreases transcription of downstream target genes		Zennaro *et al.* (1997)
MRΔ5,6		706 (75 kDa)		No evidence^a^		698 (74 kDa)		Deletion of the hinge region and the LBD		Ligand-independent; hMRΔ5,6 exerts a dominant-positive effect that enhances transcription mMRΔ5,6 does not have any effects on transcription		Zennaro *et al.* (2001), Lema *et al.* (2017)
MRIn12		988 (107 kDa)		985 (107 kDa)		984 (107 kDa)		Four-amino acid insertion at position 633		No known transcriptional function, but possibly altered DNA binding activity		Bloem *et al.* (1995)
MRΔ5		867 (94 kDa)		868 (94 kDa)		867 (94 kDa)		10-amino acid deletion of residues 672-788; deletion of the hinge region and the N-terminal part of the LBD		hMRΔ5 exerts a dominant-negative effect that decreases transcription		Arriza *et al.* (1987), Zennaro *et al.* (2001), Guo *et al.* (2020)
MRdel10		807 (86 kDa)		807 (86 kDa)		No evidence		Most of the LBD missing; 24-residue stretch at position 783 that bears no homology with the other isoforms		Ligand-independent; rMRdel10 has no intrinsic activity nor it modifies the transcriptional activity of the wildtype MR		Zhou *et al.* (2000)
MRΔ6		No evidence		No evidence		814 (86 kDa)		The LBD is truncated, and the protein terminates with a 26-residue-long C-tail		Ligand-independent; mMRΔ6 exerts a dominant-negative effect on the transcriptional activity of the wildtype MR		Lema *et al.* (2017)

a No evidence: To our knowledge the isoform has not been reported in the literature;

b Isoform Q8VII8 is missing two amino acids (positions 633 and 634 of the A3KN90 isoform). LBD, ligand-binding domain.

**Table 4:** Database information and relevant literature concerning the mineralocorticoid receptor (MR) isoforms in humans, rats, and mice.

		Transcript				Protein				
		NCBI		Ensembl		Uniprot		NCBI		Literature
**Human (*Homo sapiens*)**										
Wildtype		NM_000901.5				P08235-1 (isoform 1)		NP_000892.2		Arriza *et al.* (1987)
				ENST00000358102.8 ENST00000344721.8						
MRΔ5,6		Not listed		ENST00000342437.8		P08235-2 (isoform 2)		Not listed		Zennaro *et al.* (2001)
MRIn12		XM_011531975.1 (transcript variant X1)				P08235-3 (isoform 3)		XP_011530277.1 (isoform X1)		Bloem *et al.* (1995)
				ENST00000625323.2 ENST00000511528.1						
MRΔ5				ENST00000512865.5		P08235-4 (isoform 4)				
		NM_001354819.1						NP_001341748.1		Arriza *et al.* (1987)
		NM_001166104.2 (transcript variant 2)						NP_001159576.1 (isoform 2)		Zennaro *et al.* (2001)
MRdel10		Not listed		Not listed		Not listed		Not listed		Zhou *et al.* (2000)
MRΔ6		No evidence		No evidence		No evidence		No evidence		Absent
**Rat (*Rattus norvegicus*)**										
Wildtype		NM_013131.1		Not listed^a^		P22199-1 (isoform 1)		NP_037263.1 XP_038953454.1		Patel *et al.* (1989)
MRΔ5,6		No evidence		No evidence^b^		No evidence		No evidence		Absent
MRIn12		Not listed		ENSRNOT00000052018.5		P22199-2 (isoform 2); F1M6V2		Not listed		Bloem *et al.* (1995)
MRΔ5		No evidence		ENSRNOT00000096148.1		A0A8I6G6P1		No evidence		Arriza *et al.* (1987)
MRdel10		Not listed		Not listed		P22199-3 (isoform 3)		Not listed		Zhou *et al.* (2000)
MRΔ6		No evidence		No evidence		No evidence		No evidence		Absent
**Mouse (*Mus musculus*)**										
Wildtype										Absent^c^
		NM_001083906		ENSMUST00000109913.9		A3KN90		NP_001077375		
		XM_006530574		ENSMUST00000109912.8		Q8VII8		XP_006530637		
		XM_006530575						XP_006530638		
		XM_006530576						XP_006530639		
		XM_006530577						XP_006530640		
MRΔ5,6		Not listed		ENSMUST00000148106.8		D6RIL1		Not listed		Zennaro *et al.* (2001), Lema *et al.* (2017)
MRIn12		Not listed		ENSMUST00000034031.6		E9Q8M8		Not listed		Absent
MRΔ5		Not listed		ENSMUST00000109911.8		D3Z473		Not listed		Absent
MRdel10		No evidence		No evidence		No evidence		No evidence		Absent
MRΔ6		Not listed		Not listed		Not listed		Not listed		Lema *et al.* (2017)

a Not listed: the isoform has been reported in the literature, but it is not yet listed in the relevant database;

b No evidence: To our knowledge, the isoform has not been reported in the literature, and it is not listed in the relevant database;

c Absent: To our knowledge, the isoform has not been reported in the literature, but it is listed in the relevant database.

## Data Availability

This publication does not contain novel data. The data of the Neuro-Epigenetics Research Group at the University of Bristol, mentioned in this paper, can be found in the cited publications of this research group.
